# Solving large-scale discrete time–cost trade-off problem using hybrid multi-verse optimizer model

**DOI:** 10.1038/s41598-023-29050-9

**Published:** 2023-02-03

**Authors:** Pham Vu Hong Son, Nghiep Trinh Nguyen Dang

**Affiliations:** Department of Construction Engineering and Management, Ho Chi Minh City University of Technology (HCMUT), Vietnam National University (VNU-HCM), Ho Chi Minh City, Vietnam

**Keywords:** Civil engineering, Computational science

## Abstract

The analysis of the relationship between time and cost is a crucial aspect of construction project management. Various optimization techniques have been developed to solve time–cost trade-off problems. A hybrid multi-verse optimizer model (hDMVO) is introduced in this study, which combines the multi-verse optimizer (MVO) and the sine cosine algorithm (SCA) to address the discrete time–cost trade-off problem (DTCTP). The algorithm's optimality is evaluated by using 23 well-known benchmark test functions. The results demonstrate that hDMVO is competitive with MVO, SCA, the dragonfly algorithm and ant lion optimization. The performance of hDMVO is evaluated using four benchmark test problems of DTCTP, including two medium-scale instances (63 activities) and two large-scale instances (630 activities). The results indicate that hDMVO can provide superior solutions in the time–cost optimization of large-scale and complex projects compared to previous algorithms.

## Introduction

In project management, optimization is a highly useful tool to satisfy desired objectives under specific constraints. The productivity of different components of a project can be increased by optimization. The importance of optimization in a construction project has been emphasized for decades as it is used to find the ideal plan and schedule for completing a project. Cost optimization, time optimization, and Pareto front are three common forms of time–cost trade-off problems. The objective of the cost optimization problem is to minimize the total cost under specific conditions, including project implementation time and penalty costs for delays. Meanwhile, the time optimization problem is aimed at choosing alternative solutions to shorten the project implementation time while ensuring that the project cost does not exceed the revenue on the early operation of the project. The Pareto front is a multi-objective optimization problem to simultaneously optimize both project cost and time^1^.

Mirjalili, Mirjalili^2^ proposed a multi-verse optimizer (MVO) algorithm inspired by the Big Bang theory to satisfy the need for solving single-and multi-objective optimization problems. For result assessment, MVO is compared with other metaheuristic algorithms, such as particle swarm optimization (PSO), Genetic Algorithm (GA), Ant colony optimization (ACO), etc. The results show that the MVO algorithm can provide competitive, even superior results than those of other algorithms in most tested optimization problems. However, MVO has issues in balancing the exploration and exploitation mechanism of the search area and limitations in the search area exploitation during fast convergence, thus resulting in local optimization^3^.

The Sine Cosine Algorithm (SCA)^4^ was developed for focusing on the exploration and exploitation of the search space during optimization. The results of the test problems show that SCA can explore different regions of the search space, avoid local optimization, converge towards global optimization, and effectively exploit the promising region of the search space during optimization. In addition, the study shows that SCA converges significantly faster than PSO, GA, ACO, etc. SCA has been utilized to address optimization challenges in diverse domains since 2016^5^. Like MVO, SCA has limitations. Specifically, its search area exploitation mechanism is not clearly expressed; therefore, it easily encounters fast convergence^6^, which results in local optimization.

Two algorithms with opposite advantages and disadvantages motivated us to develop a hybrid algorithm between MVO and SCA for optimal exploration and exploitation of the search area based on the strengths of each algorithm to achieve a balance between the two mechanisms. The hDMVO algorithm was developed by preserving MVO's mechanisms of white and black holes to ensure good exploration of the search area by MVO. Concurrently, good search area exploitation by the algorithm is guaranteed by SCA through the fact that the value closest to the global optimum is stored in a variable as the target and is never lost during the optimization. Therefore, hDMVO will achieve a reasonable balance between the exploration and the exploitation phases, which ensures that the algorithm can achieve global optimization and become an appropriate metaheuristic method for solving the DTCTP.

The resolution of large-scale DTCTPs is a crucial aspect in the management of any construction project. Despite the availability of several existing methods, they are not fully equipped to solve large-scale DTCTPs. Therefore, a hybrid multi-verse optimizer model (hDMVO) was developed by combining the MVO and the SCA to provide efficient solutions for medium- and large-scale DTCTPs and other optimization problems that can be applied in actual construction projects. This model also significantly enhances the decision-making ability of decision-makers.

The rest of this paper is organized as follows. Section "Literature review" summarizes the literature on the time–cost trade-off problems. Section "Model development" outlines the development of our hybrid multi-verse optimizer model. Section "Computational experiments" presents the results from the validation and application of our model. Finally, Sections "Conclusion" and "Recommendations for future work" conclude the study and outline future research directions.

## Literature review

The stochastic optimization method is widely used in many fields of study^7,8^, which develops meta-heuristic techniques. Some popular meta-heuristic methods are inspired by animals in nature. For example, ant lion optimization (ALO) algorithm is modeled after the hunting behavior of antlions in nature. The Dragonfly algorithm (DA) is based on the static and dynamic swarming behaviors observed in dragonflies^9^. Africa Wild Dog Optimization Algorithm (AWDO) originates from the hunting mechanism of Africa wild dogs in nature^10^. Meanwhile, Genetic Algorithm (GA) is inspired by evolutionary principles, such as heredity, mutation, natural selection, and crossover^11^.

The development of new algorithms or improvement of current algorithms has recently attracted immense interest from researchers, which is related to the No Free Lunch (NFL) theorem^12^. Evidently, the NFL has enabled researchers to improve and adapt current algorithms for solving different problems or propose new algorithms to provide competitive results against current algorithms. There are a significant number of developed hybrid metaheuristic algorithms, including the ant colony system-based decision support system (ACS-SGPU)^13^, dragonfly algorithm–particle swarm optimization model^14^, quantum-based sine cosine algorithm^15^, the improved sine–cosine algorithm based on orthogonal parallel information^16^, the hybrid sine cosine algorithm with multi-orthogonal search strategy^17^.

The time–cost trade-off is extended to the discrete version, including various realistic assumptions and solved by the exact, heuristic, and metaheuristic methods. PSO and GA are metaheuristic methods commonly used in the DTCTP. Bettemir^18^ found that among eight metaheuristic methods, including a sole genetic algorithm, four hybrid genetic algorithms, PSO, ant colony optimization, and electromagnetic scatter search, PSO was one of the leading algorithms together with the hybrid genetic algorithm with quantum annealing for the large-scale cost optimization. Zhang and Xing^19^ proposed an algorithm combining PSO and fuzzy sets theory to solve the fuzzy time–cost–quality trade-off problem. Aminbakhsh and Sonmez^20^ developed the discrete particle swarm optimization method to solve the large-size time–cost trade-off problem. Aminbakhsh and Sonmez^21^ used Pareto front particle swarm optimizer (PFPSO) to simultaneously optimize the time and cost of large-scale projects. Sonmez and Bettemir^22^ presented a hybrid strategy based on GAs, simulated annealing, and quantum simulated annealing techniques for the cost optimization problem. Zhang et al.^23^ proposed a GA for the DTCTP in repetitive projects. Naseri and Ghasbeh^24^ used GA for the time–cost trade off analysis to compensate for project delays. Network analysis algorithm is also metaheuristic techniques used to solve the DTCTP^25^. Son and Khoi^26^ presented a slime mold algorithm model to solving time–cost–quality trade-off problem.

Despite their wide applications in solving the DTCTP, the metaheuristic techniques have several limitations. Therefore, hybrid metaheuristic methods are being developed and widely used in the DTCTP. An adaptive-hybrid genetic algorithm was proposed by Zheng^27^ for time–cost–quality trade-off problems. Said and Haouari^28^ developed a model wherein the simulation–optimization strategy and the mixed-integer programming formulation were used to solve the DTCTP. Tran, Luong-Duc^29^ presented an opposition multiple objective symbiotic organisms search (OMOSOS) model for time, cost, quality, and work continuity trade-off in repetitive projects. Eirgash et al.^30^ proposed a multi-objective teaching–learning-based optimization algorithm integrated with a nondominated sorting concept (NDS–TLBO), which is successfully applied to optimize the medium- to large-scale projects. A hybrid GALP algorithm combined with GA and linear programming (LP) was proposed by Alavipour and Arditi^31^ for time–cost tradeoff analysis. Albayrak^32^ developed an algorithm combining PSO and GA to solve the time–cost trade-off problem for resource-constrained construction projects. A population-based metaheuristics approach, nondominated sorting genetic algorithm III (NSGA III) was developed by Sharma and Trivedi^33^ to ensure the quality and safety in time–cost trade-off optimization. Li et al.^34^ presented an epsilon-constraint method-based genetic algorithm for uncertainty multimode time–cost–robustness trade-off problem.

This paper presents a hybrid multi-verse optimizer (hDMVO) model based on MVO and SCA, which can provide high-quality solutions for large-scale discrete time–cost trade-off optimization problems.

## Model development

### Discrete time–cost trade-off problem

The common objective of discrete time–cost tradeoff problem (DTCTP) is to minimize the total direct and indirect costs and such costs can be formulated as follows^35^:

1 $$C = min\mathop \sum \limits_{j = 1}^{S} \mathop \sum \limits_{k = 1}^{m\left( j \right)} \left( {dc_{jk} x_{jk} } \right) + D \times ic$$

subject to:

2 $$\mathop \sum \limits_{k = 1}^{m\left( j \right)} x_{jk} = 1, \forall j = \left\{ {1, \ldots ,S} \right\}$$

3 $$\mathop \sum \limits_{k = 1}^{m\left( j \right)} d_{jk} x_{ik} + St_{j} \le St_{l} , \forall l \in Sc_{j} \; and\; \forall j = \left\{ {1, \ldots ,S} \right\}$$

4 $$D \ge St_{S + 1}$$

where *C* is the project cost; *dc*_*jk*_ is the direct cost of mode *k* for activity *j*; *x*_*jk*_ is a 0–1 variable which is 1 if mode *k* is selected for executing activity *j*, and 0 otherwise; *ic* is the daily indirect cost; *D* is the project duration; *d*_*jk*_ is the duration of mode *k* for activity *j*; *St*_*j*_ is the start time for activity *j*; and *Sc*_*j*_ is the set of immediate successors for *j*.

### Hybrid multi-verse optimizer model for DTCTP

#### Multi-verse optimizer—MVO

The MVO algorithm is inspired by concepts which theoretically exist in astronomy, including white holes, black holes, and worm holes. White holes are the elements which form the universes and have been unobservable until now. Meanwhile, black holes are observable and characterized by a giant gravitational force which attracts all surrounding objects. The last element can exchange objects between different universes or different parts of a universe. In the MVO, the above three elements are mathematically modeled to develop an optimal method, simulate the teleportation and exchange of objects between universes through white/black and worm hole tunnels. In addition, the idea of the inflation of the universe is also applied to the MVO based on the inflation rate.

**Figure 1:**
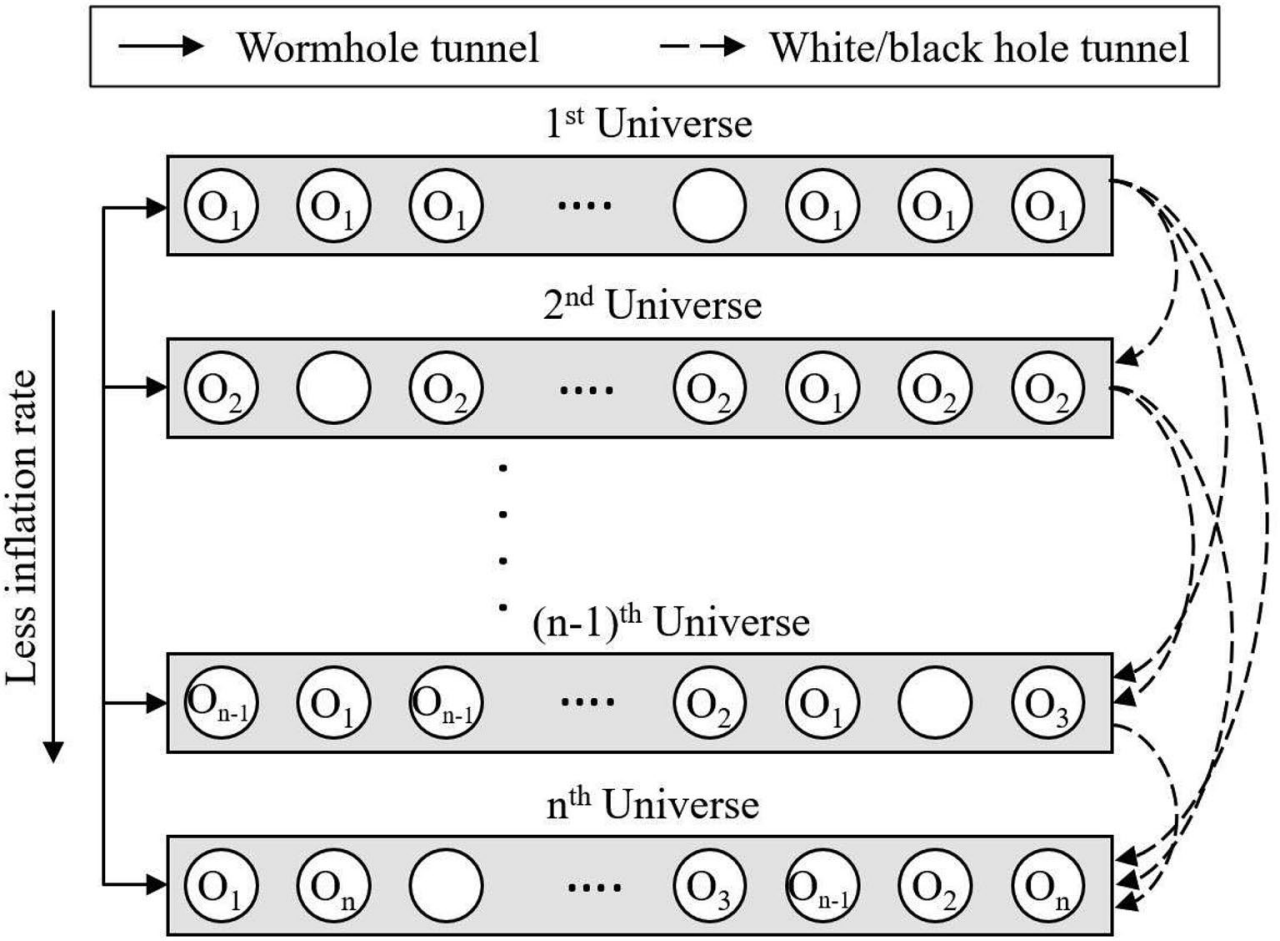
Conceptual model of the proposed MVO algorithm.

The model of the MVO algorithm is shown in Fig. 1. In this figure, the universe with a higher inflation rate will have a white hole, while a universe with a lower inflation rate will have a black hole. The objects will then be transferred from the white holes of the source universe to the black holes of the target universe. In order to improve the overall inflation rate of single universes, an assumption was made that the universes with high inflation rate would be more likely to have white holes. In contrast, the universes with low inflation rate are more likely to have black holes. In Fig. 1, the white points represent celestial bodies travelling through the worm holes. It can be observed that worm holes stochastically change celestial bodies regardless of their inflation rates.

The roulette wheel mechanism will be used. (Eq. 6) to mathematically model white or black hole tunnels and exchange celestial objects between universes. When optimization problems are solved with the maximized objective function, –NI will be changed into NI. In each iteration, universes will be rearranged based on their inflation rate (fitness value), and by the roulette wheel mechanism, one universe will be selected in the occurrence of white hole, assume that:

5 $$U = \left[ {\begin{array}{*{20}c} {\begin{array}{*{20}c} {x_{1}^{1} } & {x_{1}^{2} } \\ \end{array} } & \ldots & {x_{1}^{d} } \\ {\begin{array}{*{20}c} {x_{2}^{1} } & {x_{2}^{2} } \\ \end{array} } & \ldots & {x_{2}^{d} } \\ {\begin{array}{*{20}c} {\begin{array}{*{20}c} \ldots \\ {x_{n}^{1} } \\ \end{array} } & {\begin{array}{*{20}c} \ldots \\ {x_{n}^{2} } \\ \end{array} } \\ \end{array} } & {\begin{array}{*{20}c} \ldots \\ \ldots \\ \end{array} } & {\begin{array}{*{20}c} \ldots \\ {x_{n}^{d} } \\ \end{array} } \\ \end{array} } \right]$$

where *d* is the number of parameters (variables) and *n* is the number of universes (candidate solutions):

6 $$x_{i}^{j} = \left\{ {\begin{array}{*{20}c} {x_{k}^{j} r_{1} < NI\left( {U_{i} } \right)} \\ {x_{i}^{j} r_{1} \ge NI\left( {U_{i} } \right)} \\ \end{array} } \right.$$

where $x_{i}^{j}$ indicates the *j*th parameter of *i*th universe, *U*_*i*_ shows the *i*th universe, *NI*(*U*_*i*_) is normalized inflation rate of the *i*th universe, *r*_1_ is a random number in [0, 1], and ${x}_{k}^{j}$ indicates the *j*th parameter of *k*th universe selected by a roulette wheel selection mechanism.

With the above-mentioned mechanism, universes keep the objects exchanged without interference. For accurate determination of the diversity of universes and exploitation, each universe has a wormhole to stochastically transport its objects through space. In order to provide local changes for each universe and improve inflation rate by using wormholes, worm hole tunes are assumed to always be established between a universe and a best universe formed so far. This mechanism is presented as follows:

7 $$x_{i}^{j} = \left\{ {\begin{array}{*{20}c} {\left\{ {\begin{array}{*{20}c} {X_{J} + TDR \times \left( {\left( {ub_{j} - lb_{j} } \right) \times r_{4} + lb_{j} } \right) r_{3} < 0.5} \\ {X_{J} - TDR \times \left( {\left( {ub_{j} - lb_{j} } \right) \times r_{4} + lb_{j} } \right) r_{3} \ge 0.5} \\ \end{array} r_{2} < WEP} \right.} \\ {x_{i}^{j} r_{2} \ge WEP} \\ \end{array} } \right.$$

where *X*_*j*_ indicates the *j*th parameter of best universe formed so far, *TDR* is a coefficient, *WEP* is another coefficient, *lb*_*j*_ shows the lower bound of *j*th variable, *ub*_*j*_ is the upper bound of *j*th variable, ${x}_{i}^{j}$ indicates the *j*th parameter of *i*th universe, and *r*_2_, *r*_3_, *r*_4_ are random numbers in [0, 1].

Two main coefficients, namely the wormhole existence probability (*WEP*) and travelling distance rate (*TDR*) can be seen in Eq. (7). The coefficient *WEP* was used to determine the wormhole existence probability in the universe. Such coefficient will linearly increase over the iterations (Eq. 8).

8 $$WEP = min + l \times \left( { \frac{max - min}{L}} \right)$$

where the *min* variable is the minimum value, the *max* variable is the maximum value, *l* presents the number of the current iteration, and *L* presents the termination criteria (the maximum number of iterations).

**Figure 2:**
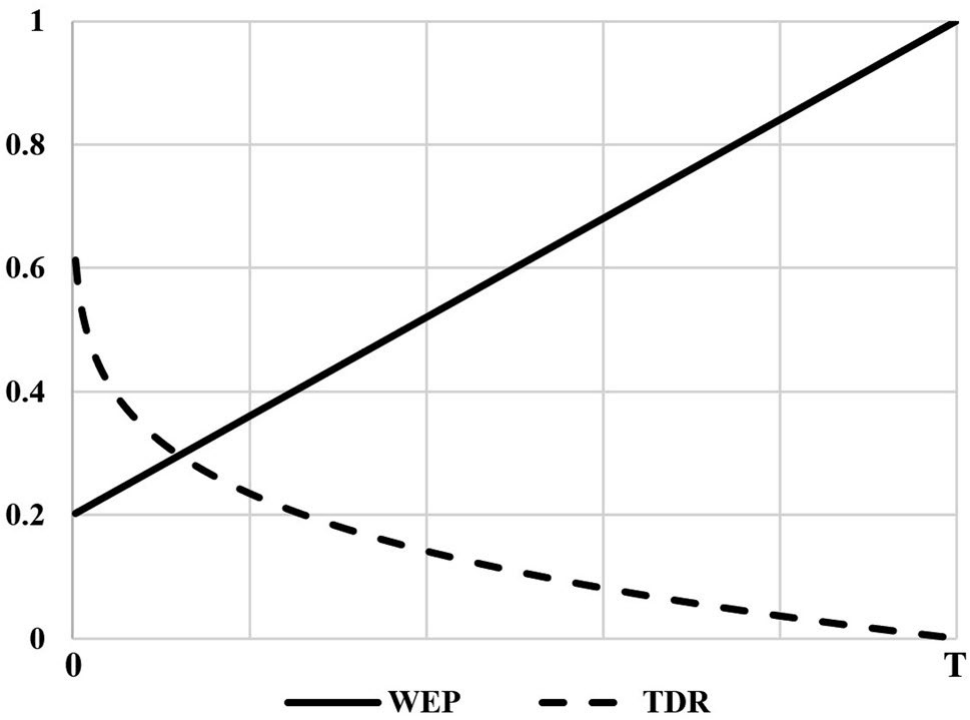
Wormhole existence probability (WEP) versus travelling distance rate (TDR).

*TDR* is a factor to determine the distance rate (variation) by which an object can be displaced by a wormhole around the best universe formed so far. (Eq. 9). In contrast to *WEP*, *TDR* decreases over iterations for more precise exploitation or local search around the best universe formed so far (Fig. 2).

9 $$TDR = 1 - \frac{{l^{1/p} }}{{L^{1/p} }}$$

where *p* presents the exploitation rate through the iterations. The larger *p*, the earlier and more precise exploitation/local search.

In the MVO algorithm, the optimization process starts with generating a set of random universes. At each iteration, objects in universes with higher inflation rates tend to travel to universes with lower inflation rates through white or black holes. Meanwhile, every universe has to face random processes of celestial bodies through wormholes to reach the best universe. This process is repeated until the termination criteria are satisfied (such as a predetermined maximum number of iterations).

#### Sine cosine algorithm-SCA

Stochastic population-based techniques have in common is to divide the optimization process into two phases: exploration and exploitation^36^. In the exploration phase, the optimization algorithm will abruptly combine solutions with a high random rate to find the promising region of the search space. However, in the exploitation phase, there will be gradual changes in the stochastic solutions, and the stochastic variations will be significantly less than those in the exploration phase.

In SCA, the mathematical equations for updating positions are given for both phases, see Eqs. (10) and (11):

10 $$X_{j}^{t + 1} = X_{j}^{t} + \alpha_{1} \times \sin \left( {\alpha_{2} } \right) \times \left| {\alpha_{3} D_{j}^{t} - X_{j}^{t} } \right|$$

11 $$X_{j}^{t + 1} = X_{j}^{t} + \alpha_{1} \times \cos \left( {\alpha_{2} } \right) \times \left| {\alpha_{3} D_{j}^{t} - X_{j}^{t} } \right|$$

where $X_{j}^{t}$ is the position of the current solution in *i*th dimension at *t*th iteration, *α*_1_, *α*_2_ and *α*_3_ are random numbers, $D_{j}^{t}$ is position of the destination point in *i*th dimension, and || indicates the absolute value.

The above two formulas are combined into a general formula as follows:

12 $$X_{j}^{t + 1} = \left\{ {\begin{array}{*{20}c} {X_{j}^{t} + \alpha_{1} \times \sin \left( {\alpha_{2} } \right) \times \left| {\alpha_{3} P_{j}^{t} - X_{j}^{t} } \right| \alpha_{4} < 0.5} \\ {X_{j}^{t} + \alpha_{1} \times \cos \left( {\alpha_{2} } \right) \times \left| {\alpha_{3} P_{j}^{t} - X_{j}^{t} } \right| \alpha_{4} \ge 0.5} \\ \end{array} } \right.$$

where *α*_*4*_ is a random number in [0,1].

In Eq. (12), it can be seen that SCA has 4 main parameters: *α*_1_, *α*_2_, *α*_3_, and *α*_4_. *α*_1_ defines the movement direction, *α*_2_ determines how far the movement should be towards or outwards the destination, *α*_3_ denotes random weights for destination. Finally, the parameter *α*_4_ switches between the sine and cosine components in Eq. (12).

**Figure 3:**
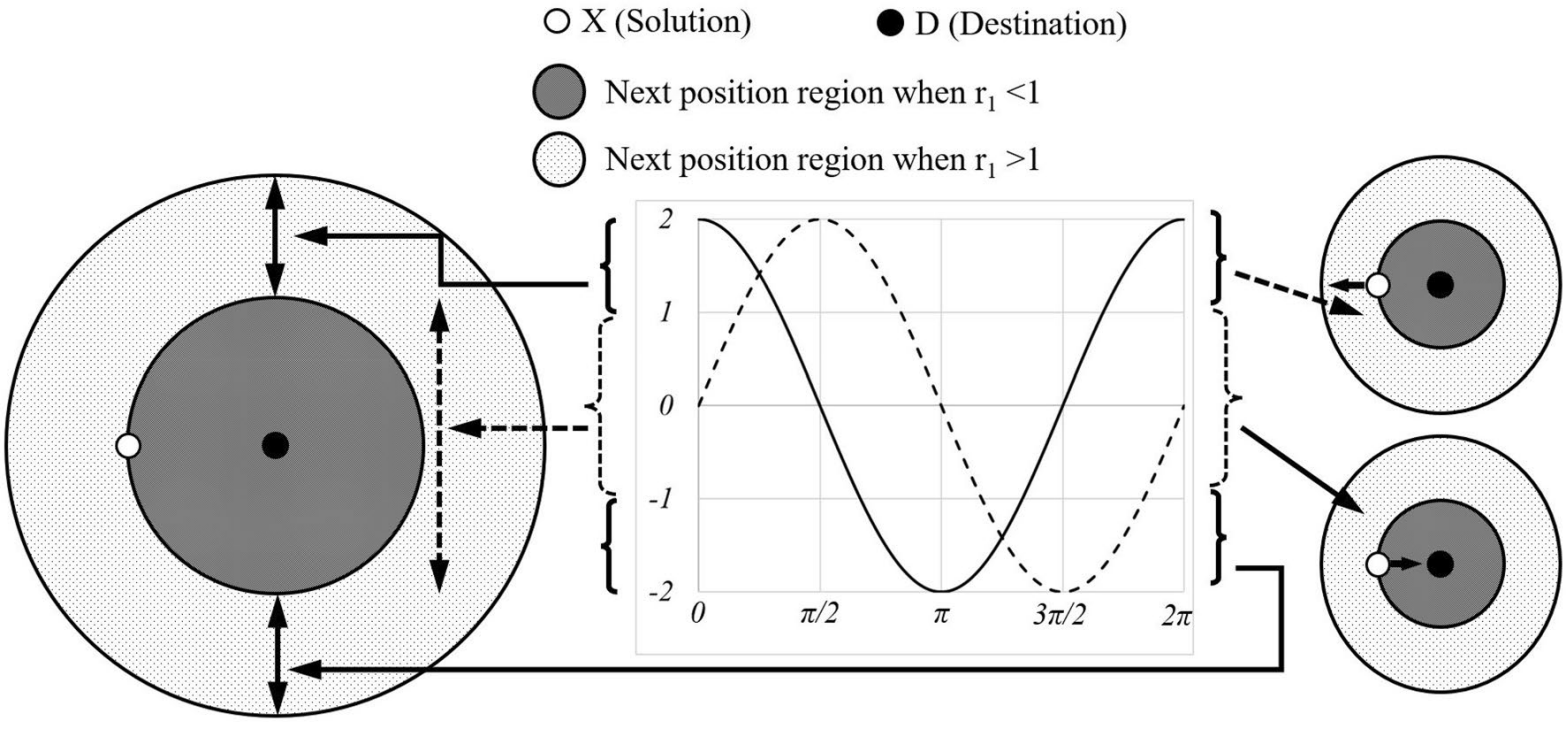
Sine and cosine with the range in [− 2, 2] allow a solution to go around (inside the space between them) or beyond (outside the space between them) the destination.

A general model in Fig. 3 shows the effectiveness of the sine and cosine functions in the range [− 2, 2]. This figure shows how the range of sine and cosine changes in order to update the location of a response. Randomness is also achieved by determining a random number for *α*_2_ in [0, 2π] (Eq. (12)). Therefore, this mechanism ensures the exploration of the search space.

**Figure 4:**
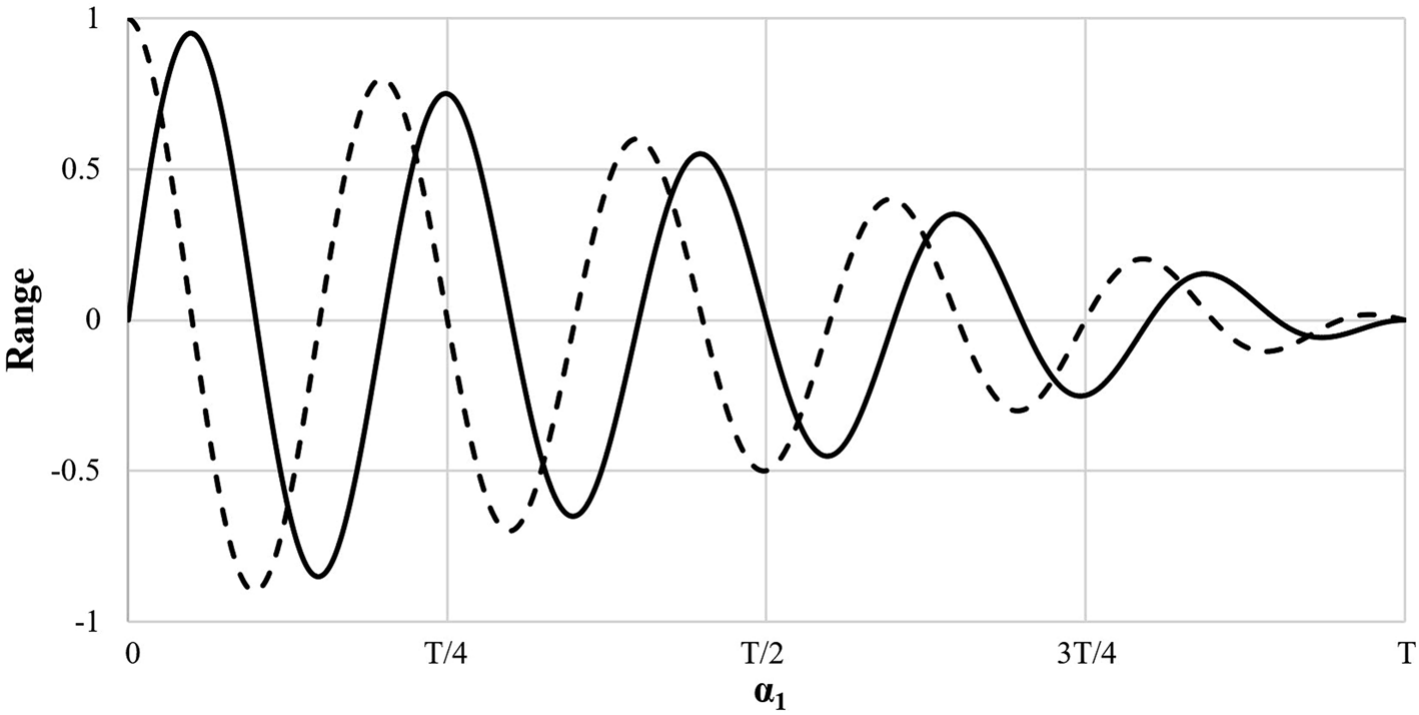
Decreasing pattern for range of sine and cosine.

In each iteration, the range of Sine and Cosine functions in Eqs. (10)–(12) will be changed to balance the exploitation and exploration phases in order to find the promising regions of the search space and finally achieve the global optimization by Eq. (13):

13 $$\alpha_{1} = v - t\frac{v}{T}$$

where *v* is a constant, *t* is the current iteration and *T* is the maximum number of iterations. Figure 4 shows the reduction in the range of the sine and cosine functions over the course of iterations.

#### Hybrid multi-verse optimizer model for DTCTP

By taking advantage of SCA and MVO, the hDMVO is built to change the MVO's exploitation mechanism by the SCA's exploitation mechanism while preserving the MVO’s mechanisms of roulette wheel selection Eq. (14), thereby improving the hDMVO's search area exploration and exploitation.

14 $$x_{i}^{j} = \left\{ {\begin{array}{*{20}c} {x_{k}^{j} \delta_{1} < NI\left( {U_{i} } \right)} \\ {x_{i}^{j} \delta_{1} \ge NI\left( {U_{i} } \right)} \\ \end{array} } \right.$$

where ${x}_{i}^{j}$ indicates the *j*th parameter of *i*th universe, *U*_*i*_ shows the *i*th universe, *NI*(*U*_*i*_) is normalized inflation rate of the *i*th universe, *δ*_1_ is a random number in [0, 1], and ${x}_{k}^{j}$ indicates the *j*th parameter of *k*th universe selected by a roulette wheel selection mechanism.

**Figure 5:**
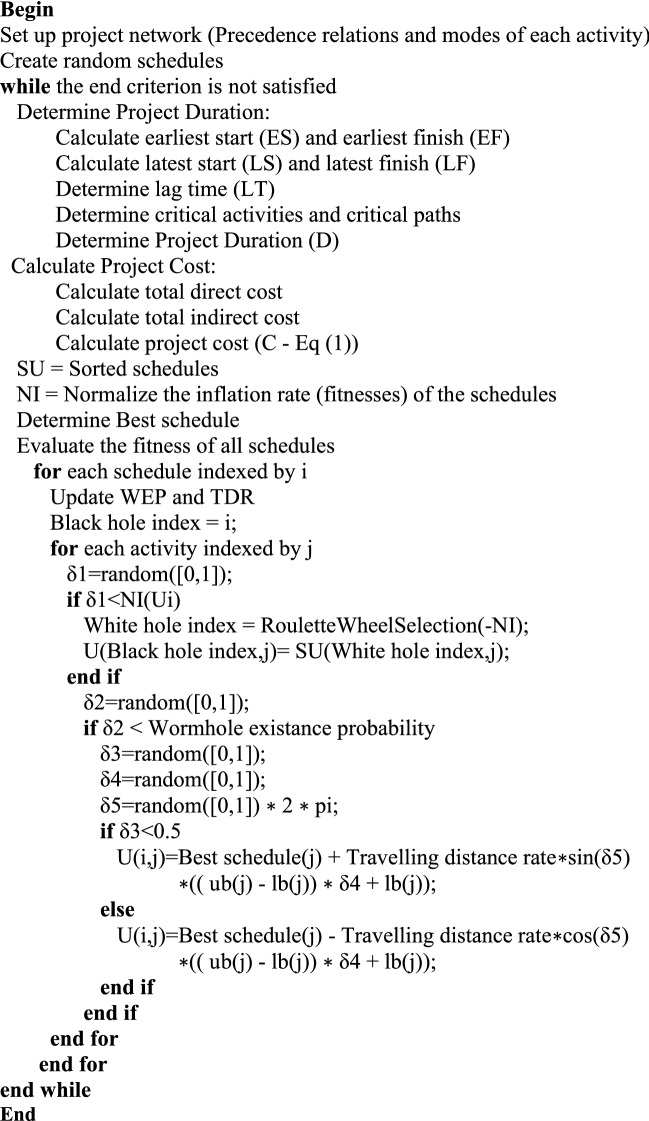
Pseudo-code of the proposed hDMVO algorithm.

**Figure 6:**
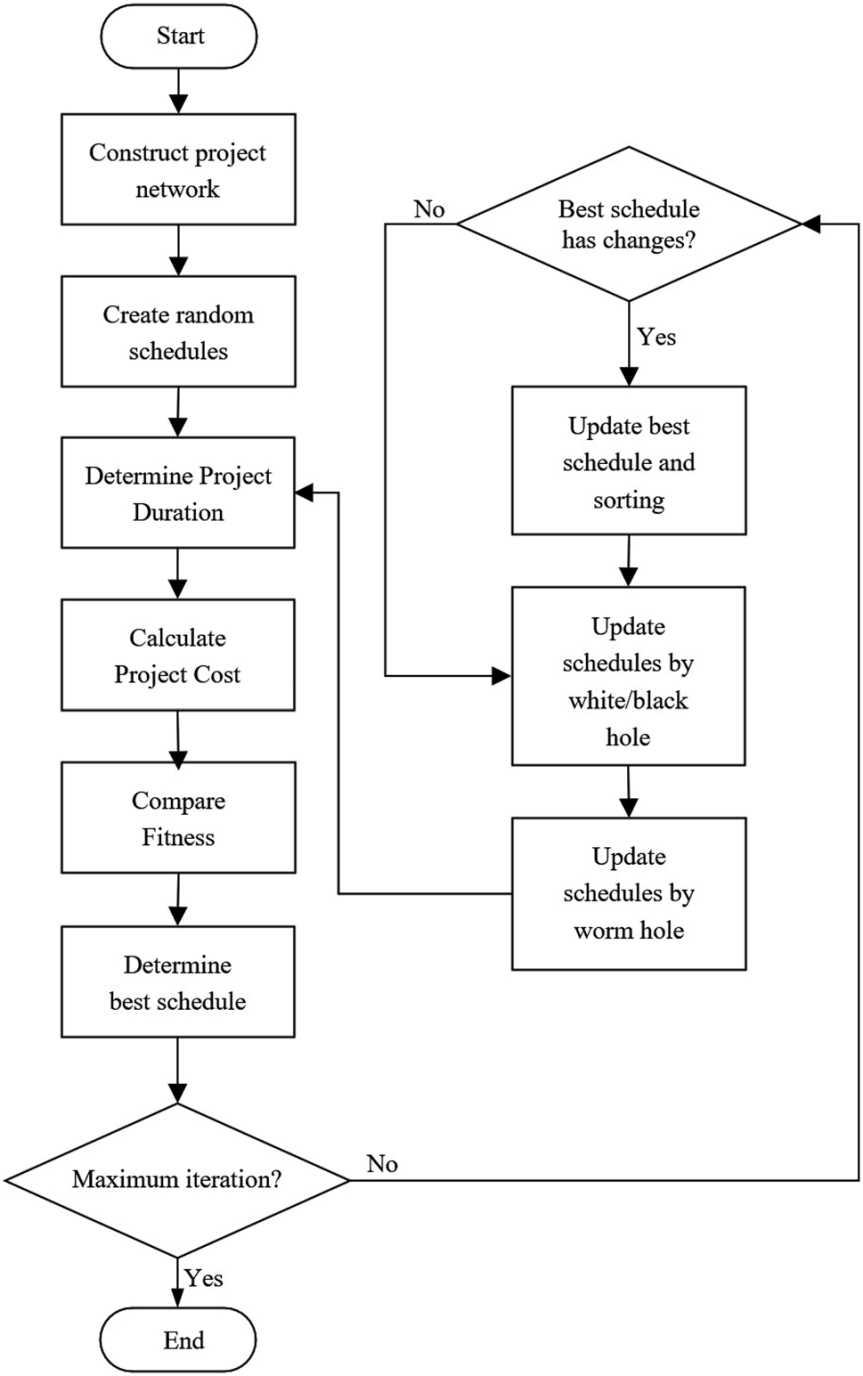
Flowchart of the proposed hDMVO algorithm.

A new formula which combines two algorithms MVO and SCA will be developed from Eq. (9) and Eq. (12) as follows:

15 $$x_{i}^{j} = \left\{ {\begin{array}{*{20}c} {\left\{ {\begin{array}{*{20}c} {X_{J} + TDR \times \sin \left( {\delta_{5} } \right) \times \left( {\left( {ub_{j} - lb_{j} } \right) \times \delta_{4} + lb_{j} } \right) \delta_{3} < 0.5} \\ {X_{J} - TDR \times \cos \left( {\delta_{5} } \right) \times \left( {\left( {ub_{j} - lb_{j} } \right) \times \delta_{4} + lb_{j} } \right) \delta_{3} \ge 0.5} \\ \end{array} \delta_{2} < WEP} \right.} \\ {x_{i}^{j} \delta_{2} \ge WEP} \\ \end{array} } \right.$$

where *X*_*j*_ indicates the *j*th parameter of best universe formed so far. *TDR* is wormhole existence probability was calculated by Eq. (8) with *min* = 0.2 and *max* = 3. *WEP* is travelling distance rate was calculated by Eq. (9) with *p* = 10. *lb*_*j*_ shows the lower bound of *j*th variable, *ub*_*j*_ is the upper bound of *j*th variable, ${x}_{i}^{j}$ indicates the *j*th parameter of *i*th universe, and *δ*_2_, *δ*_3_, *δ*_4_ are random numbers in [0, 1]. *δ*_5_ are also random numbers in [0, 2π]. The parameter *δ*_5_ defines how far the movement should be towards or outwards the destination. The pseudo-code and flowchart of our hDMVO method is given in Figs. 5 and 6. The set of parameters that are summarized in Table 1 provided an adequate combination for the hDMVO, MVO and SCA.

**Table 1:** Parameter settings of the hDMVO, SCA and MVO.

Algorithm	Parameter	Description	Value
hDMVO	N	Number of iterations	250
	i	Number of solutions	200
	min	Minimum value	0.2
	max	Maximum value	3
	p	Exploitation rate	10
	δ_1_	Random number	[0, 1]
	δ_2_	Random number	[0, 1]
	δ_3_	Random number	[0, 1]
	δ_4_	Random number	[0, 1]
	δ_5_	Random number	[0, 2π]
SCA	N	Number of iterations	250
	i	Number of solutions	200
	v	Constant value	3
	α_2_	Random number	[0, 2π]
	α_3_	Random number	[0, 2]
	α_4_	Random number	[0, 1]
MVO	N	Number of iterations	250
	i	Number of solutions	200
	min	Minimum value	0.2
	max	Maximum value	3
	p	Exploitation rate	10
	r_1_	Random number	[0, 1]
	r_2_	Random number	[0, 1]
	r_3_	Random number	[0, 1]
	r_4_	Random number	[0, 1]

The complexity of the hDMVO algorithm is influenced by various factors such as the number of activities, schedules, iterations, roulette wheel selection mechanism, and sorting mechanism. The roulette wheel selection method, which is applied for every activity in each solution over the course of iterations, has a complexity of O(log N). The sorting of solutions is carried out at each iteration by utilizing the Quicksort algorithm, which has a complexity of O(N^2) in the worst-case scenario. As a result, the overall computational complexity is:

16 $$O\left( {hDMVO} \right) = O\left( {T\left( {O\left( {Quicksort\, algorithm} \right) + N \times n \times \left( {O\left( {roulette\, wheel\, selection} \right)} \right)} \right)} \right)$$

17 $$O\left( {hDMVO} \right) = O\left( {T\left( {N^{2} + N \times n \times \log N} \right)} \right)$$

where n is the number of activities, N is the number of schedules, and T is the maximum iterations.

In the optimization process, the solution *a* is evaluated to be better than solution *b* if:

18 $$a > b\, if\, C_{a} < C_{b}$$

In case the project cost is equal (*C*_*a*_ = *C*_*b*_), The option with the shortest completion time is considered the best one:

19 $$a > b\, if \left\{ {\begin{array}{*{20}c} {C_{a} = C_{b} } \\ {D_{a} < D_{b} } \\ \end{array} } \right.$$

In case both options a and b have the same project cost and project duration, the optimal solution will be stochastically selected.

The exploration and exploitation of the search space will be significantly improved thanks to the hDMVO algorithm. Such hybrid algorithm not only searches for the optimal solution from the sets of solutions stochastically generated at the initial phase, but can also exploit the space between solutions through each iteration to find new promising regions of the search space.

## Computational experiments

### Convergence behaviours

To assess the optimization capabilities of hDMVO, a comprehensive analysis was conducted using twenty-three well-known benchmark test functions. Comparisons were made to the results obtained from four other optimization algorithms, including MVO, SCA, DA, and ALO. These benchmark functions are grouped into three categories: unimodal, multimodal, and fixed-dimension multi-modal functions, as detailed in Tables 2, 3 and 4.

**Table 2:** Uni-modal test functions.

Funtion	Dim	Range	fmin
$f1\left(x\right)={\sum }_{i=1}^{n}{x}_{i}^{2}$	10	[− 100, 100]	0
$f2\left(x\right)={\sum }_{i=1}^{n}\left|{x}_{i}\right|+ {\prod }_{i=1}^{n}\left|{x}_{i}\right|$	10	[− 10, 10]	0
$f3\left(x\right)={\sum }_{i=1}^{n}{\left({\sum }_{j-1}^{i}{x}_{j}\right)}^{2}$	10	[− 100, 100]	0
$f4\left(x\right)=max\left\{\left|{x}_{i}\right|, 1 \le i \le n\right\}$	10	[− 100, 100]	0
$f5\left(x\right)={\sum }_{i=1}^{n-1}\left[100{\left({x}_{i+1}-{x}_{i}^{2}\right)}^{2}+{({x}_{i}-1)}^{2}\right]$	10	[− 30, 30]	0
$f6\left(x\right)={\sum }_{i=1}^{n}{(|{x}_{i}+0.5|)}^{2}$	10	[− 100, 100]	0
$f7\left(x\right)={\sum }_{i=1}^{n}i{x}_{i}^{4}+random[\mathrm{0,1})$	10	[− 1.28, 1.28]	0

**Table 3:** Multi-modal test functions.

Funtion	Dim	Range	fmin
$f8\left(x\right)={\sum }_{i=1}^{n}-{x}_{i}\mathrm{sin}(\sqrt{|{x}_{i}|})$	10	[− 500, 500]	–2820.8
$f9\left(x\right)={\sum }_{i=1}^{n}[{x}_{i}^{2}-10\mathrm{cos}\left(2\pi {x}_{i}\right)+10]$	10	[− 5.12, 5.12]	0
$f10\left(x\right)=-20\mathrm{exp}\left(-0.2\sqrt{\frac{1}{n}{\sum }_{i=1}^{n}{x}_{i}^{2}}\right)-exp\left(\frac{1}{n}{\sum }_{i=1}^{n}\mathrm{cos}\left(2\pi {x}_{i}\right)\right)+20+e$	10	[− 32, 32]	0
$f11\left(x\right)=\frac{1}{4000}{\sum }_{i=1}^{n}{x}_{i}^{2}-{\prod }_{i=1}^{n}\mathrm{cos}\left(\frac{{x}_{i}}{\sqrt{i}}\right)+1$	10	[− 600, 600]	0
$$f12\left(x\right)=\frac{\pi }{n}\left\{10{\mathrm{sin}}^{2}(\pi {y}_{1})+{\sum }_{i=1}^{n}{({y}_{i}-1)}^{2}\left[1+10{\mathrm{sin}}^{2}(\pi {y}_{i+1})\right]+{({y}_{n}-1)}^{2}+{\sum }_{i=1}^{n}u({x}_{i},\mathrm{10,100,4})\right\}$$			
$${y}_{i}=1+\frac{{x}_{i}+1}{4}$$			
$$u\left({x}_{i},a,k,m\right)=\left\{\begin{array}{c}k{\left({x}_{i}-a\right)}^{m} {x}_{i}>a\\ 0 -a< {x}_{i}<a\\ k{\left({-x}_{i}-a\right)}^{m} {x}_{i}<-a\end{array}\right.$$	10	[− 50, 50]	0
$f13\left(x\right)=0.1\left\{{\mathrm{sin}}^{2}\left(3\pi {x}_{1}\right)+{\sum }_{i=1}^{n}{\left({x}_{i}-1\right)}^{2}\left[1+{\mathrm{sin}}^{2}\left(3\pi {x}_{i}+1\right)\right]+{\left({x}_{n}-1\right)}^{2}[1+{\mathrm{sin}}^{2}(2\pi {x}_{n})]\right\}+{\sum }_{i=1}^{n}u({x}_{i},\mathrm{5,100,4})$	10	[− 50, 50]	0

**Table 4:** Fixed-dimension multi-modal test functions.

Funtion	Dim	Range	fmin
$f14\left(x\right)={\left(\frac{1}{500}+{\sum }_{j=1}^{25}\frac{1}{j+{\sum }_{i=1}^{2}{\left({x}_{i}-{a}_{ij}\right)}^{6}}\right)}^{-1}$	2	[− 65, 65]	1
$f15\left(x\right)={\sum }_{i=1}^{11}{\left[{a}_{i}-\frac{{x}_{1}({b}_{i}^{2}+{b}_{i}{x}_{2})}{{b}_{i}^{2}+{b}_{i}{x}_{3}+{x}_{4}}\right]}^{2}$	4	[− 5, 5]	0.0003
$f16\left(x\right)=4{x}_{1}^{2}-2.1{x}_{1}^{4}+\frac{1}{3}{x}_{1}^{6}+{x}_{1}{x}_{2}-4{x}_{2}^{2}+4{x}_{2}^{4}$	2	[− 5, 5]	–1.0316
$f17\left(x\right)={\left({x}_{2}-\frac{5.1}{4{\pi }^{2}}{x}_{1}^{2}+\frac{5}{\pi }{x}_{1}-6\right)}^{2}+10\left(1-\frac{1}{8\pi }\right)cos{x}_{1}+10$	2	[− 5, 5]	0.398
$f18\left(x\right)=\left[1+{\left({x}_{1}+{x}_{2}+1\right)}^{2}(19-14{x}_{1}+3{x}_{1}^{2}-14{x}_{2}+6{x}_{1}{x}_{2}+3{x}_{2}^{2})\right]\times \left[30+{(2{x}_{1}-3{x}_{2})}^{2}\times \left(18-32{x}_{1}+12{x}_{1}^{2}+48{x}_{2}-36{x}_{1}{x}_{2}+27{x}_{2}^{2}\right)\right]$	2	[− 2, 2]	3
$f19\left(x\right)=-{\sum }_{i=1}^{4}{c}_{i}exp\left(-{\sum }_{j=1}^{3}{a}_{ij}{({x}_{j}-{p}_{ij})}^{2}\right)$	3	[0, 1]	–3.86
$f20\left(x\right)=-{\sum }_{i=1}^{4}{c}_{i}exp\left(-{\sum }_{j=1}^{6}{a}_{ij}{({x}_{j}-{p}_{ij})}^{2}\right)$	6	[0, 1]	–3.32
$f21\left(x\right)=-{\sum }_{i=1}^{5}{\left[\left(X-{a}_{i}\right){\left(X-{a}_{i}\right)}^{T}+{c}_{i}\right]}^{-1}$	4	[0, 10]	–10.1532
$f22\left(x\right)=-{\sum }_{i=1}^{7}{\left[\left(X-{a}_{i}\right){\left(X-{a}_{i}\right)}^{T}+{c}_{i}\right]}^{-1}$	4	[0, 10]	–10.4028
$f23\left(x\right)=-{\sum }_{i=1}^{10}{\left[\left(X-{a}_{i}\right){\left(X-{a}_{i}\right)}^{T}+{c}_{i}\right]}^{-1}$	4	[0, 10]	–10.5363

In order to ensure a fair comparison, all of the algorithms were executed 30 times for each benchmark function. Statistical results, including the mean value (ave) and standard deviation (std), were collected from 30 runs of the algorithm. It is important to note that 30 search agents and a maximum of 500 iterations were utilized in the analysis. The statistical results of the hDMVO algorithm, as well as those of other comparative algorithms (DA, ALO, SCA, and MVO), can be found in Tables 5, 6 and 7.

**Table 5:** Results of unimodal benchmark functions.

F	hDMVO		MVO		SCA		DA		ALO	
	ave	std	ave	std	ave	std	ave	std	ave	std
F1	1.588E−03	1.791E−03	1.519E−02	4.661E−03	2.966E−11	1.148E−10	1.550E+01	2.913E+01	1.183E−05	6.369E−06
F2	9.787E−03	2.555E−03	4.339E−02	9.607E−03	6.977E−09	6.947E−09	1.359E+00	1.178E+00	9.683E+00	1.039E+01
F3	1.160E−02	2.206E−02	1.714E−01	1.128E−01	6.278E−01	3.267E+00	4.137E+02	9.523E+02	7.644E+02	3.653E+02
F4	2.507E−02	1.137E−02	1.203E−01	3.002E−02	5.852E−03	1.203E−02	3.041E+00	1.730E+00	1.003E+01	3.991E+00
F5	5.334E+00	1.243E+00	4.746E+02	7.199E+02	1.511E+01	2.943E+01	2.476E+03	9.311E+03	1.029E+03	7.344E+02
F6	1.287E−03	1.038E−03	2.321E−02	6.481E−03	6.067E−01	8.437E−02	1.220E+01	2.244E+01	1.088E−05	8.070E−06
F7	7.937E−04	2.949E−04	5.013E−03	1.613E−03	7.475E−03	3.299E−03	2.417E−02	1.674E−02	2.157E−01	4.782E−02

**Table 6:** Results of multi-modal benchmark functions.

F	hDMVO		MVO		SCA		DA		ALO	
	ave	std	ave	std	ave	std	ave	std	ave	std
F8	− 3.296E+03	1.413E+02	− 2.631E+03	1.657E+02	− 1.996E+03	6.324E+01	− 2.713E+03	3.334E+02	− 1.881E+03	5.375E+01
F9	1.181E+01	2.604E+00	2.027E+01	4.340E+00	3.362E+00	5.921E+00	2.606E+01	1.043E+01	4.786E+01	6.938E+00
F10	1.510E−02	4.330E−03	5.682E−01	6.412E−01	8.207E−01	2.255E+00	2.854E+00	1.487E+00	8.339E+00	4.557E+00
F11	1.485E−01	3.619E−02	4.592E−01	7.921E−02	2.346E−01	2.252E−01	5.682E−01	3.300E−01	2.977E−01	9.121E−02
F12	2.252E−05	8.048E−06	1.550E−01	2.116E−01	1.278E−01	2.571E−02	1.992E+00	1.539E+00	1.156E+01	3.978E+00
F13	1.410E−04	1.363E−04	9.961E−03	5.517E−03	4.077E−01	4.464E−02	1.840E+00	3.217E+00	2.218E−02	2.141E−02

**Table 7:** Results of composite benchmark functions.

F	hDMVO		MVO		SCA		DA		ALO	
	ave	std	ave	std	ave	std	ave	std	ave	std
F14	9.980E−01	6.206E−13	9.980E−01	4.146E−11	3.241E+00	1.397E+00	1.229E+00	7.947E−01	1.099E+01	3.772E+00
F15	5.491E−04	1.321E−04	1.314E−02	1.486E−02	1.515E−03	7.317E−05	5.974E−03	8.523E−03	1.535E−02	1.233E−02
F16	− 1.032E+00	3.428E−09	− 1.032E+00	2.912E−07	− 1.032E+00	8.055E−05	− 1.032E+00	9.266E−06	− 1.004E+00	1.465E−01
F17	3.979E−01	2.395E−09	3.979E−01	6.596E−07	4.020E−01	2.509E−03	3.979E−01	1.336E−05	3.979E−01	4.733E−13
F18	3.000E+00	5.107E−08	5.700E+00	1.454E+01	3.000E+00	1.130E−04	3.000E+00	1.215E−05	3.000E+00	2.638E−12
F19	− 3.863E+00	1.333E−08	− 3.863E+00	2.664E−06	− 3.852E+00	1.728E−03	− 3.863E+00	2.644E−04	− 3.863E+00	3.780E−06
F20	− 3.322E+00	1.905E−07	− 3.201E+00	2.178E−03	− 2.507E+00	5.012E−01	− 3.265E+00	7.245E−02	− 3.206E+00	4.805E−02
F21	− 1.015E+01	3.950E−05	− 4.098E+00	1.195E+00	− 7.773E−01	1.688E−01	− 7.351E+00	2.616E+00	− 2.658E+00	2.614E−02
F22	− 1.040E+01	1.488E−05	− 8.047E+00	2.955E+00	− 1.168E+00	5.738E−01	− 7.083E+00	2.990E+00	− 3.054E+00	6.087E−01
F23	− 1.036E+01	9.708E−01	− 6.657E+00	3.304E+00	− 1.892E+00	8.817E−01	− 7.517E+00	3.315E+00	− 2.534E+00	2.465E−01

It should be noted that unimodal functions possess a single global optimum, making them an appropriate choice for evaluating the exploitation mechanism. An examination of the results presented in Table 5 reveals that hDMVO exhibited superior exploitability when compared to other swarm-based optimization algorithms (DA, ALO, SCA, and MVO) in the unimodal test functions, as demonstrated by its performance in 7 out of 7 for MVO and DA, 5 out of 7 for ALO and 4 out of 7 for SCA.

Unlike unimodal functions, multimodal benchmark functions possess a global optimization point in addition to many local optima. Therefore, multimodal test functions are well-suited for evaluating the exploration capabilities of hDMVO. The results for multimodal test functions (Table 6) show that hDMVO performs better than MVO, DA and ALO, and is comparable to SCA (6 out of 6 for MVO, ALO and DA, 5 out of 6 for SCA). Therefore, the ability of hDMVO to effectively avoid local optima and explore the search space has been demonstrated through its performance.

The composite test functions, as the name implies, are a combination of various unimodal and multimodal test functions, which include variations such as rotation, shifting, and bias. These composite test functions have a similar real search space with multiple local optima, which is beneficial for testing the balance between exploration and exploitation of the search space. The results of the hDMVO algorithm's performance with composite test functions (F14–F23) are presented in Table 7. The results indicate that the hDMVO outperforms other population-based optimization algorithms in terms of average values, thus demonstrating its ability to effectively balance search space exploration and exploitation.

**Figure 7:**
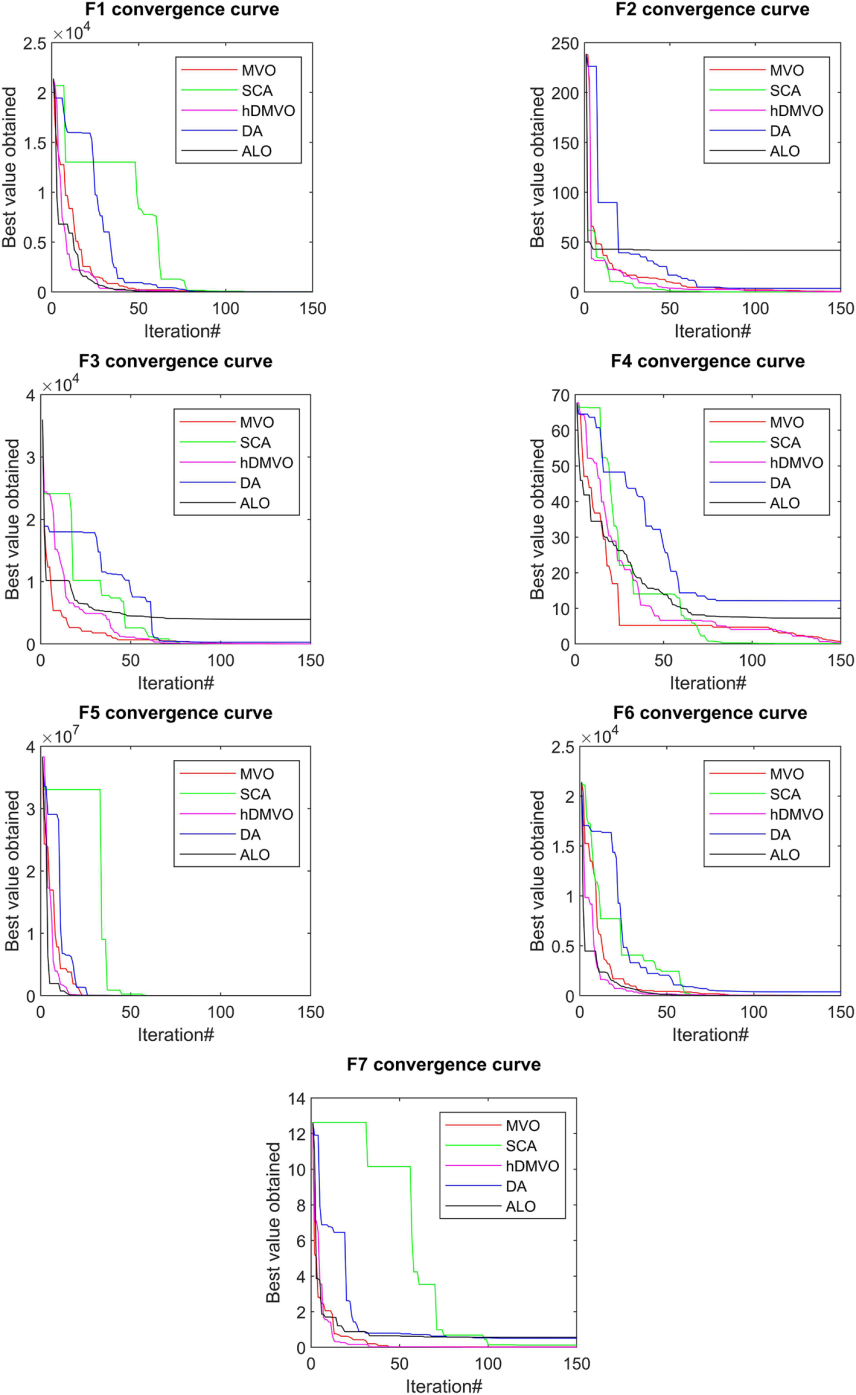
Convergence curves of MVO, SCA, ALO, DA, and hDMVO variants for unimodal functions.

**Figure 8:**
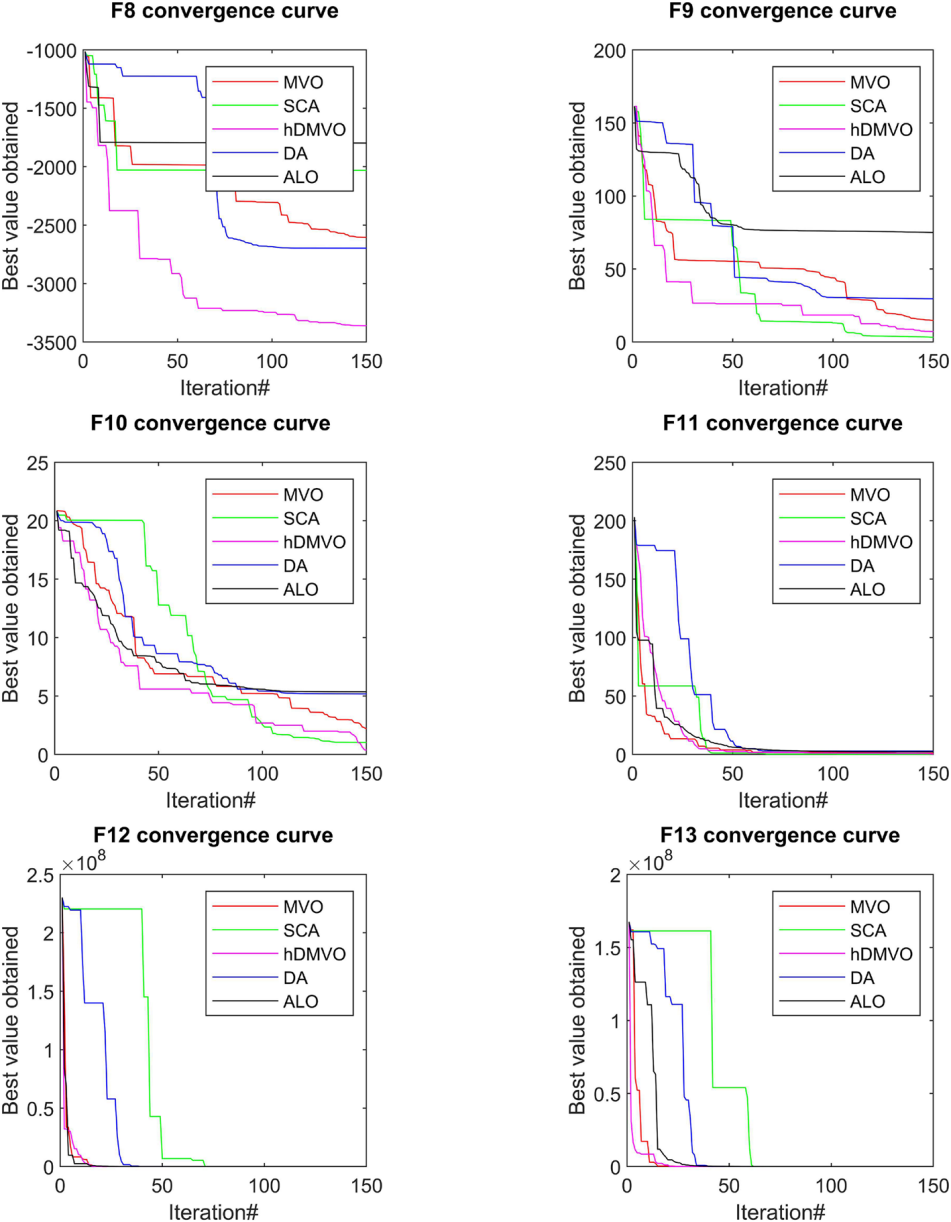
Convergence curves of MVO, SCA, ALO, DA, and hDMVO variants for multimodal functions.

**Figure 9:**
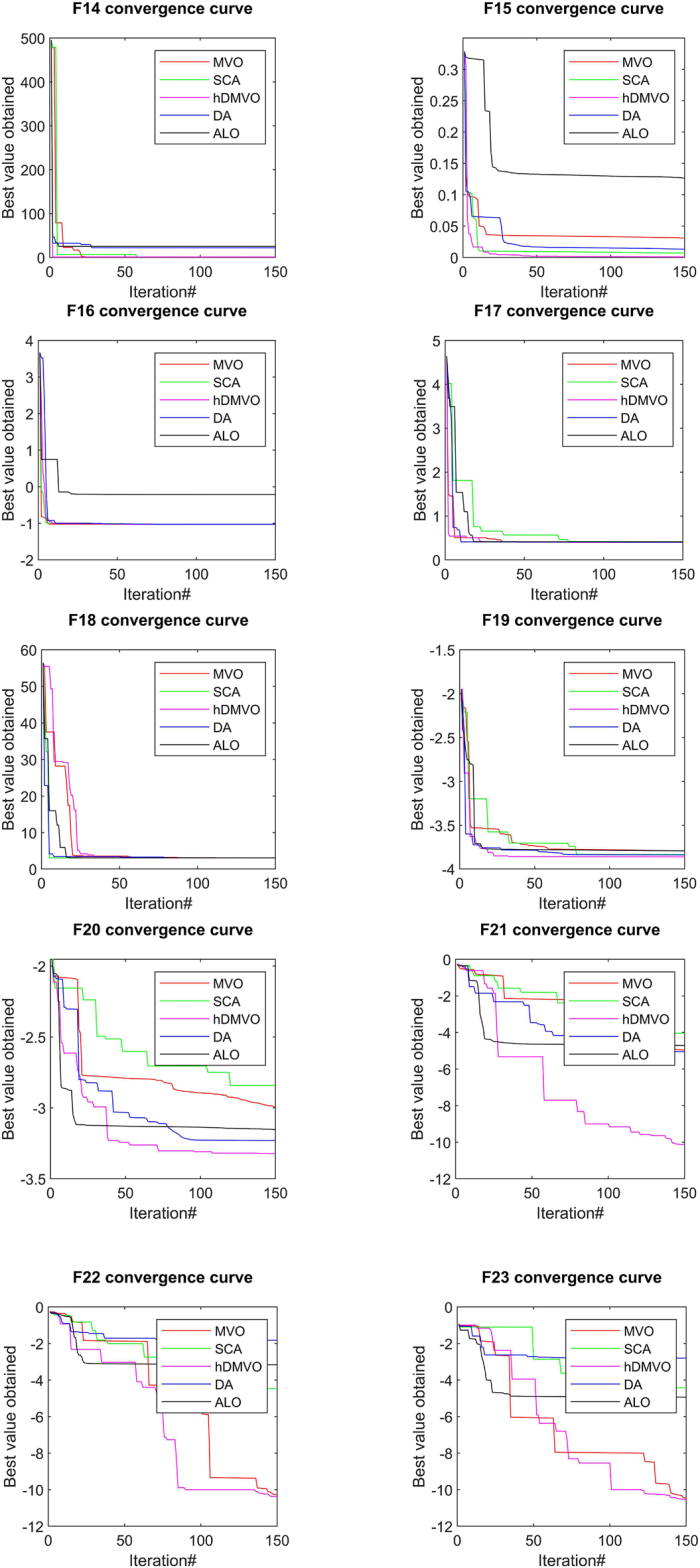
Convergence curves of MVO, SCA, ALO, DA, and hDMVO variants for composite functions.

The performance of the hDMVO algorithm in terms of convergence, in comparison to other state-of-the-art algorithms (DA, ALO, SCA, and MVO), is depicted in Figs. 7, 8 and 9. Through the use of 10 agents and 150 iterations, the study generated convergence curves which demonstrate that hDMVO has a higher likelihood of reaching optimal convergence on a majority of the benchmark test functions.

### Medium-scale instances of DTCTP

**Figure 10:**
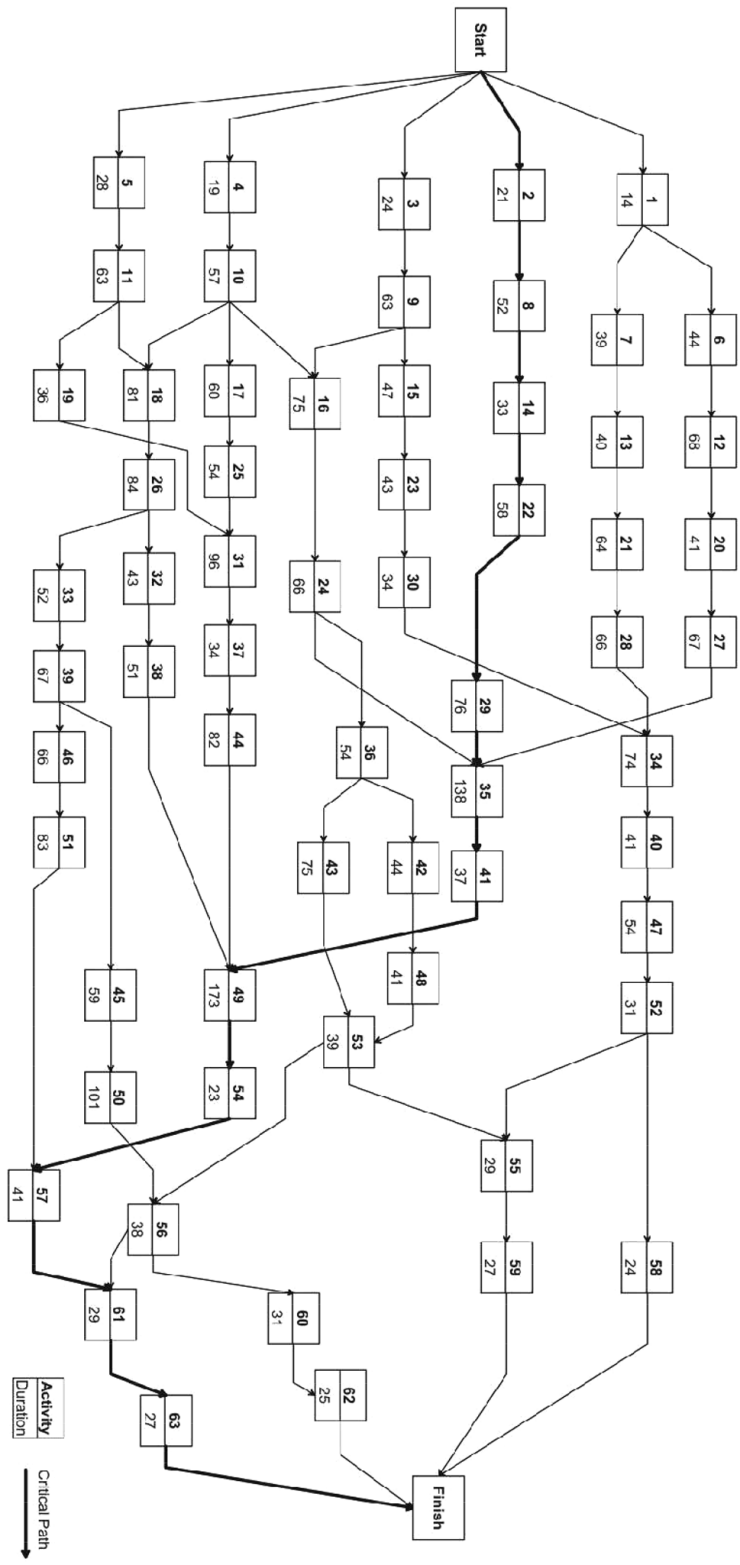
Activity on the node (AoN) representation of the 63-activity network (Aminbakhsh and Sonmez^20^).

The medium-scale instances include two 63-activity problems wherein each activity has maximum five modes^22^. The network diagram of this problem is shown in Fig. 10. The time–cost alternatives for these instances are listed in Table 8. The medium-scale instances, including 1.37 × 10^42^ possible solutions will be tested at two different levels of indirect costs. The indirect cost in the first problem (63a) is 2300USD/day, while that in the second problem (63b) is 3500USD/day. The optimal solutions for these two problems are 5,421,120USD and 6,176,170USD, respectively. The hDMVO algorithm has been implemented in Python and is compatible with Visual Studio Code. The testing of all instances of DTCTP were performed on a personal computer featuring an Intel Core i7-8750H 2.20 GHz CPU and 8.0 GB of RAM.

**Table 8:** Data for the 63 activity time–cost trade-off problem.

Action	Pred	Dur1	Cost1	Dur2	Cost2	Dur3	Cost3	Dur4	Cost4	Dur5	Cost5
1	–	14	3750	12	4250	10	5400	9	6250	–	–
2	–	21	11,250	18	14,800	17	16,200	15	19,650	–	–
3	–	24	22,450	22	24,900	19	27,950	17	31,650	–	–
4	–	19	17,800	17	19,400	15	21,600	–	–	–	–
5	–	28	31,180	26	34,200	23	38,250	21	41,400	–	–
6	1	44	54,260	42	58,450	38	63,225	35	68,150	–	–
7	1	39	47,600	36	50,750	33	54,800	30	59,750	–	–
8	2	52	62,140	47	69,700	44	72,600	39	81,750	–	–
9	3	63	72,750	59	79,450	55	86,250	51	91,500	49	99,500
10	4	57	66,500	53	70,250	50	75,800	46	80,750	41	86,450
11	5	63	83,100	59	89,450	55	97,800	50	104,250	45	112,400
12	6	68	75,500	62	82,000	58	87,500	53	91,800	49	96,550
13	7	40	34,250	37	38,500	33	43,950	31	48,750	–	–
14	8	33	52,750	30	58,450	27	63,400	25	66,250	–	–
15	9	47	38,140	40	41,500	35	47,650	32	54,100	–	–
16	9,10	75	94,600	70	101,250	66	112,750	61	124,500	57	132,850
17	10	60	78,450	55	84,500	49	91,250	47	94,640	–	–
18	10,11	81	127,150	73	143,250	66	154,600	61	161,900	–	–
19	11	36	82,500	34	94,800	30	101,700	–	–	–	–
20	12	41	48,350	37	53,250	34	59,450	32	66,800	–	–
21	13	64	85,250	60	92,600	57	99,800	53	107,500	49	113,750
22	14	58	74,250	53	79,100	50	86,700	47	91,500	42	97,400
23	15	43	66,450	41	69,800	37	75,800	33	81,400	30	88,450
24	16	66	72,500	62	78,500	58	83,700	53	89,350	49	96,400
25	17	54	66,650	50	70,100	47	74,800	43	79,500	40	86,800
26	18	84	93,500	79	102,500	73	111,250	68	119,750	62	128,500
27	20	67	78,500	60	86,450	57	89,100	56	91,500	53	94,750
28	21	66	85,000	63	89,750	60	92,500	58	96,800	54	100,500
29	22	76	92,700	71	98,500	67	104,600	64	109,900	60	115,600
30	23	34	27,500	32	29,800	29	31,750	27	33,800	26	36,200
31	19,25	96	145,000	89	154,800	83	168,650	77	179,500	72	189,100
32	26	43	43,150	40	48,300	37	51,450	35	54,600	33	61,450
33	26	52	61,250	49	64,350	44	68,750	41	74,500	38	79,500
34	28,30	74	89,250	71	93,800	66	99,750	62	105,100	57	114,250
35	24,27,29	138	183,000	126	201,500	115	238,000	103	283,750	98	297,500
36	24	54	47,500	49	50,750	42	56,800	38	62,750	33	68,250
37	31	34	22,500	32	24,100	29	26,750	27	29,800	24	31,600
38	32	51	61,250	47	65,800	44	71,250	41	76,500	38	80,400
39	33	67	81,150	61	87,600	57	92,100	52	97,450	49	102,800
40	34	41	45,250	39	48,400	36	51,200	33	54,700	31	58,200
41	35	37	17,500	31	21,200	27	26,850	23	32,300	–	–
42	36	44	36,400	41	39,750	38	42,800	32	48,300	30	50,250
43	36	75	66,800	69	71,200	63	76,400	59	81,300	54	86,200
44	37	82	102,750	76	109,500	70	127,000	66	136,800	63	146,000
45	39	59	84,750	55	91,400	51	101,300	47	126,500	43	142,750
46	39	66	94,250	63	99,500	59	108,250	55	118,500	50	136,000
47	40	54	73,500	51	78,500	47	83,600	44	88,700	41	93,400
48	42	41	36,750	39	39,800	37	43,800	34	48,500	31	53,950
49	38,41,44	173	267,500	159	289,700	147	312,000	138	352,500	121	397,750
50	45	101	47,800	74	61,300	63	76,800	49	91,500	–	–
51	46	83	84,600	77	93,650	72	98,500	65	104,600	61	113,200
52	47	31	23,150	28	27,600	26	29,800	24	32,750	21	35,200
53	43,48	39	31,500	36	34,250	33	37,800	29	41,250	26	44,600
54	49	23	16,500	22	17,800	21	19,750	20	21,200	18	24,300
55	52,53	29	23,400	27	25,250	26	26,900	24	29,400	22	32,500
56	50,53	38	41,250	35	44,650	33	47,800	31	51,400	29	55,450
57	51,54	41	37,800	38	41,250	35	45,600	32	49,750	30	53,400
58	52	24	12,500	22	13,600	20	15,250	18	16,800	16	19,450
59	55	27	34,600	24	37,500	22	41,250	19	46,750	17	50,750
60	56	31	28,500	29	30,500	27	33,250	25	38,000	21	43,800
61	56,57	29	22,500	27	24,750	25	27,250	22	29,800	20	33,500
62	60	25	38,750	23	41,200	21	44,750	19	49,800	17	51,100
63	61	27	9500	26	9700	25	10,100	24	10,800	22	12,700

**Figure 11:**
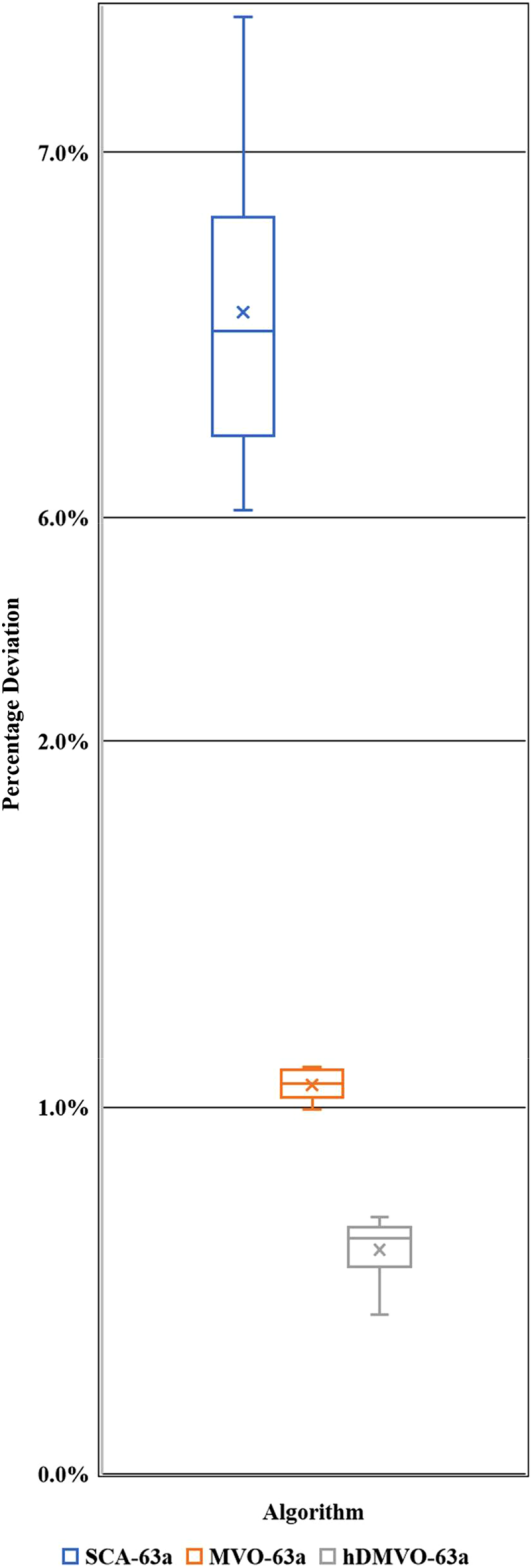
Percentage deviations of hDMVO, MVO and SCA in problem 63a.

**Figure 12:**
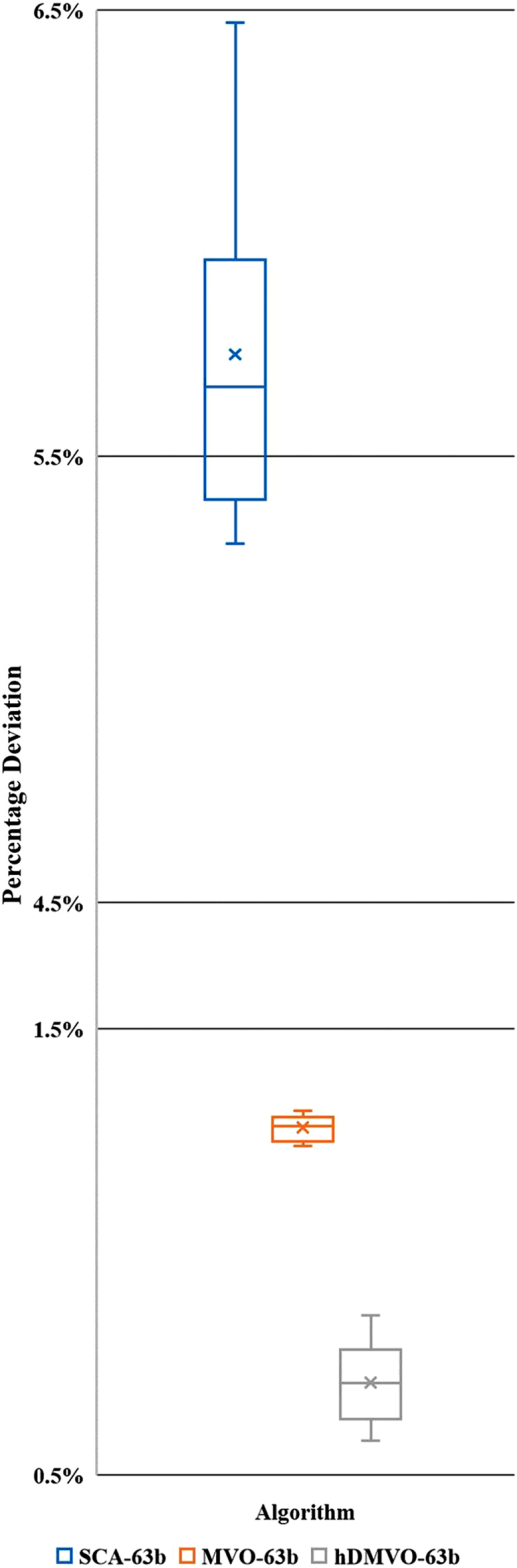
Percentage deviations of hDMVO, MVO and SCA in problem 63b.

hDMVO obtains exceptional results in medium-scale experimental problems, wherein the best values among the ten runs are 5,444,670USD for problems 63a (Table 9) and 6,211,720USD for problems 63b (Table 10). The distribution of percentage deviations for hDMVO, MVO, and SCA are illustrated in Figs. 11 and 12, using ten trials of the 63a and 63b DTCTP problems. The figures demonstrate that hDMVO has a smaller deviation percentage compared to MVO and SCA, indicating its ability to effectively balance exploration and exploitation when solving medium-scale DTCTP problems.

**Table 9:** Analysis results of problem 63a.

No	SCA		MVO		hDMVO	
	Dur	Cost	Dur	Cost	Dur	Cost
1	656	5,747,490	635	5,475,030	632	5,444,670
2	631	5,748,170	629	5,475,980	638	5,445,380
3	626	5,761,900	637	5,476,980	635	5,453,820
4	638	5,772,130	631	5,477,170	626	5,455,050
5	638	5,772,330	612	5,478,790	635	5,455,620
6	650	5,775,665	636	5,478,930	629	5,456,190
7	632	5,783,255	636	5,479,820	636	5,457,050
8	652	5,788,430	633	5,480,820	630	5,457,270
9	628	5,798,400	632	5,481,190	632	5,458,260
10	629	5,820,580	630	5,481,280	651	5,459,130
Pop. size	200		200		200	
Num. of iterations	250		250		250	
Num. of function evaluation	50,000		50,000		50,000

**Table 10:** Analysis results of problem 63b.

No	SCA		MVO		hDMVO	
	Dur	Cost	Dur	Cost	Dur	Cost
1	628	6,524,960	596	6,252,550	626	6,211,720
2	623	6,515,560	621	6,252,790	592	6,213,340
3	607	6,507,315	602	6,253,410	613	6,214,290
4	601	6,534,900	595	6,254,850	613	6,216,270
5	602	6,525,940	626	6,255,250	618	6,219,720
6	588	6,510,775	623	6,255,420	614	6,219,780
7	618	6,532,965	586	6,256,265	623	6,223,840
8	614	6,567,555	598	6,256,440	621	6,224,580
9	617	6,503,725	602	6,256,790	590	6,225,290
10	571	6,575,900	627	6,257,390	619	6,229,130
Pop. size	200		200		200	
Num. of iterations	250		250		250	
Num. of function evaluation	50,000		50,000		50,000

The average percent deviation (APD) of hDMVO from the global optimal for problems 63a and 63b is summarized in Table 11. The results show that hDMVO outperforms ACO, GA, and electromagnetism mechanism (EMS)^18^, sole genetic algorithm (GA), hybrid genetic algorithm (HA)^22^, modified adaptive weight approach with genetic algorithms (MAWA-GA), modified adaptive weight approach with particle swarm optimization (MAWA-PSO) and modified adaptive weight approach with teaching learning based optimization (MAWA-TLBO)^37^. hDMVO also outperforms the two original algorithms named MVO and SCA when its ADPs are 0.61% and 0.71% for problems 63a and 63b, respectively, within 50,000 schedules. As evident from Table 11, hDMVO outperforms both native algorithms (MVO and SCA) in medium-scale instances. By searching only 50,000 solutions out of 1.37 × 10^42^ potential solutions, hDMVO can identify high-quality solutions that were highly close to the optimal value.

**Table 11:** Average percent deviation from the optimal for problem 63a and 63b.

Algorithm	63a		63b	
	No. of runs	APD (%)	No. of runs	APD (%)
ACO (Bettemir^18^)	10	1.30	10	0.80
EMS (Bettemir^18^)	10	2.13	10	2.40
GA (Bettemir^18^)	10	5.19	10	4.27
GA (Sonmez and Bettemir^22^)	10	5.86	10	5.16
HA (Sonmez and Bettemir^22^)	10	2.61	10	2.50
MAWA-GA (Toğan and Eirgash^37^)	10	7.01	10	4.07
MAWA-PSO (Toğan and Eirgash^37^)	10	8.38	10	7.72
MAWA-TLBO (Toğan and Eirgash^37^)	10	3.62	10	1.63
SCA (This study)	10	6.56	10	5.73
MVO (This study)	10	1.06	10	1.28
hDMVO (This study)	10	0.61	10	0.71

### Large-scale instances of DTCTP

The large-scale instances include two 630-activity problems, wherein each activity has a maximum of five modes, including 2.38 × 10^421^ possible solutions^22^. These cases represent the size of an actual construction project. The indirect costs for large-scale instances (630a and 630b) are similar to those of medium-scale instances (2300USD/day and 3500USD/day for 63a and 63b, respectively).

**Figure 13:**
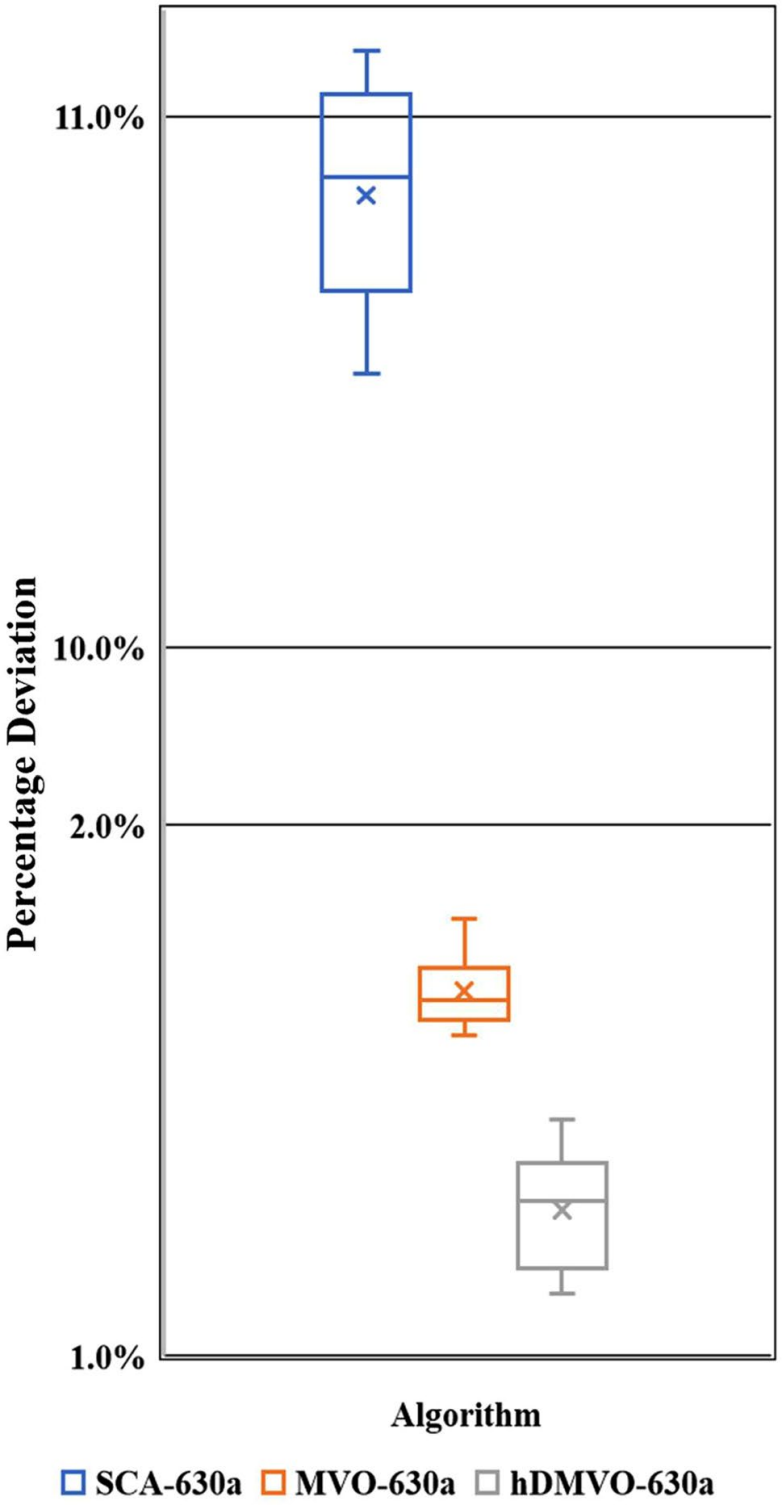
Percentage deviations of hDMVO, MVO and SCA in problem 630a.

**Figure 14:**
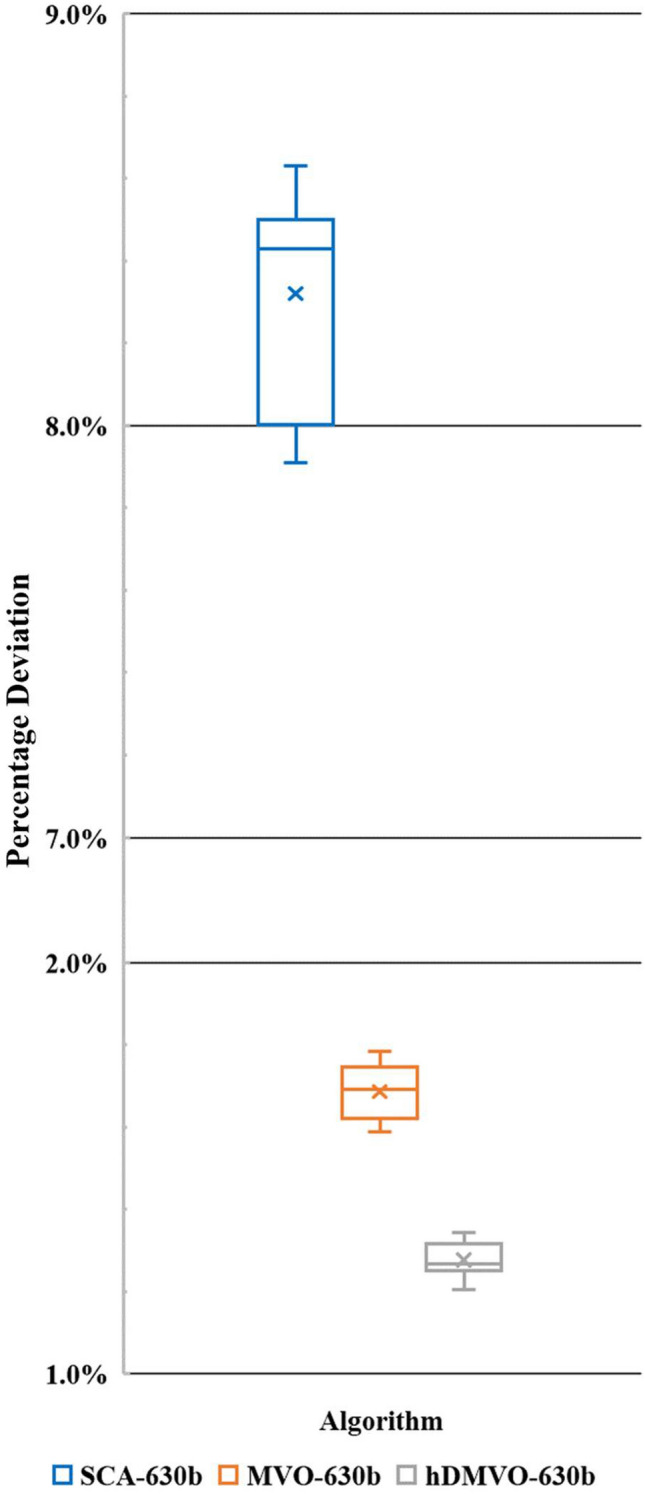
Percentage deviations of hDMVO, MVO and SCA in problem 630b.

The hDMVO algorithm also shows its effectiveness in large-scale experimental problems; the best values for ten runs in problems 630a and 630b are 54,816,950USD (Table 12) and 62,505,580USD (Table 13), respectively. Figures 13 and 14 show the boxplots of ten percentage deviations of hDMVO, MVO and SCA by testing problems 630a and 630b. From Figs. 13 and 14, the percentage deviations of hDMVO are much smaller than those of MVO and SCA. The results thus demonstrated the stability of hDMVO when solving the large-scale DTCTP problem.

**Table 12:** Analysis results of problem 630a.

No	SCA		MVO		hDMVO	
	Dur	Cost	Dur	Cost	Dur	Cost
1	5654	59,911,250	6323	55,080,630	6317	54,816,950
2	5628	59,980,560	6291	55,082,810	6306	54,820,585
3	5615	60,000,610	6322	55,099,890	6330	54,849,050
4	5612	60,019,530	6296	55,110,705	6300	54,883,485
5	5641	60,082,860	6295	55,111,040	6324	54,897,700
6	5613	60,141,055	6270	55,122,000	6311	54,924,330
7	5632	60,165,040	6273	55,122,360	6322	54,937,240
8	5631	60,196,945	6281	55,135,870	6339	54,949,000
9	5612	60,197,985	6313	55,190,530	6355	54,951,200
10	5586	60,240,730	6334	55,199,825	6291	54,993,930
Pop. size	200		200		200	
Num. of iterations	250		250		250	
Num. of function evaluation	50,000		50,000		50,000

**Table 13:** Analysis results of problem 630b.

No	SCA		MVO		hDMVO	
	Dur	Cost	Dur	Cost	Dur	Cost
1	5604	66,647,315	6052	62,742,405	6097	62,505,580
2	5592	66,676,865	6088	62,759,350	5991	62,526,385
3	5587	66,712,740	5997	62,763,350	6020	62,537,900
4	5597	66,924,805	6027	62,783,920	6117	62,538,830
5	5583	66,961,860	6064	62,798,565	6042	62,544,040
6	5632	66,975,580	6027	62,815,320	6056	62,544,470
7	5642	66,985,310	6016	62,827,245	6105	62,561,260
8	5604	67,006,655	6036	62,839,590	6015	62,570,390
9	5599	67,026,155	5985	62,843,840	5998	62,588,460
10	5609	67,092,830	6097	62,863,315	6060	62,591,215
Pop. size	200		200		200	
Num. of iterations	250		250		250	
Num. of function evaluation	50,000		50,000		50,000

For large-scale instances, hDMVO provides superior results (Table 14) against GA, genetic algorithm with simulated annealing (GASA), hybrid genetic algorithm with quantum simulated annealing (HGAQSA), genetic memetic algorithm with simulated annealing (GMASA), genetic algorithm with simulated annealing and variable neighborhood search (GASAVNS), PSO, electromagnetic scatter search (ESS)^18^, and GA and HA^22^. hDMVO completely outperforms SCA and performs slightly better than MVO. hDMVO also yields better results than NDS–TLBO^30^ in problem 630b; for problem 630a, hDMVO achieves APD of 1.27% when searching for 50,000 solutions, while NDS–TLBO achieves APD of 1.1% when searching for 250,000 solutions. hDMVO's ADP values for problems 630a and 630b are 1.27% and 1.28%, respectively (Table 8), which were significantly superior to those of the two original algorithms, MVO and SCA. By just searching for 50,000 solutions out of 2.38 × 10^421^ potential solutions, hDMVO can achieve high-quality solutions for large-scale instances. These results indicate that hDMVO has overcome the disadvantages of MVO and SCA in search space exploration and exploitation to achieve the optimal value.

**Table 14:** Average percent deviation from the optimal for problem 630a and 630b.

Algorithm	630a		630b	
	No. of runs	APD (%)	No. of runs	APD (%)
GA (Bettemir^18^)	10	8.83	10	7.50
GASA (Bettemir^18^)	10	8.84	10	7.59
HGAQSA (Bettemir^18^)	10	2.41	10	2.47
GMASA (Bettemir^18^)	10	8.02	10	7.56
GASAVNS (Bettemir^18^)	10	7.97	10	7.38
PSO (Bettemir^18^)	10	1.12	10	2.20
ACO (Bettemir^18^)	10	4.95	10	4.56
ESS (Bettemir^18^)	10	8.86	10	7.70
GA (Sonmez and Bettemir^22^)	10	8.83	10	7.50
HA (Sonmez and Bettemir ^22^)	10	2.41	10	2.47
NDS-TLBO (Eirgash, Toğan et al.^30^)	10	1.1	10	1.51
SCA (This study)	10	10.85	10	8.32
MVO (This study)	10	1.69	10	1.69
hDMVO (This study)	10	1.27	10	1.28

## Conclusion

This study presents a combined model of MVO and SCA for global optimization. The combination's objective is to make use of the exploration of MVO and the search space exploitation of SCA to achieve an effective balance between the two phases during optimization. hDMVO is developed to combine the search space exploitation mechanism of MVO and SCA while preserving MVO's mechanisms of roulette wheel selection, thereby improving hDMVO's search exploration and exploitation. hDMVO was comprehensively evaluated by twenty-three benchmark optimization problems. The results indicate that hDMVO is more likely to achieve global optimization compared with SCA and MVO. In this study, hDMVO is proposed to solve the discrete time–cost trade-off problem in construction projects. The results of the computational experiments reveal that hDMVO can achieve high-quality solutions for medium- and large-scale DTCTP and can be used to optimize the cost–time problems for actual projects. With the obtained results, hDMVO is seen as an appropriate metaheuristic method for solving the DTCTP problem as well as other optimization problems.

## Recommendations for future work

In this study, the application of hDMVO is limited to solving DTCTP problems with the finish-to-start relationship. However, in actual construction projects, DTCTP problems in construction projects often include the start-to-start, finish-to-finish, and start-to-finish relationships. Therefore, in future studies, hDMVO will be used to solve DTCTP problems with complicated relationships simultaneously and on a large scale to obtain more comprehensive solutions for project management. The hDMVO model has been shown to effectively balance exploration and exploitation when compared to other state-of-the-art swarm-based optimization algorithms (DA, ALO, SCA, and MVO). Additionally, the hDMVO model also demonstrates competitive performance in medium- and large-scale DTCTPs. However, limitations in local optima avoidance are also clearly demonstrated by hDMVO when applied to large-scale problems. To overcome these limitations, future research will involve the development of a combination model that incorporates hDMVO with other techniques such as modified adaptive weight approach and opposition-based learning, to enhance its performance in solving optimization problems in the construction industry and other technical fields.

## Data Availability

Some or all data, models, or code that support the findings of this study are available from the corresponding author upon reasonable request.
